# Comparison of adherence measurement tools used in a pre-exposure prophylaxis demonstration study among female sex workers in Benin

**DOI:** 10.1097/MD.0000000000020063

**Published:** 2020-05-22

**Authors:** Aminata Mboup, Luc Béhanzin, Fernand Guédou, Katia Giguère, Nassirou Geraldo, Djimon Marcel Zannou, René K. Kêkê, Moussa Bachabi, Flore Gangbo, Dissou Affolabi, Mark A. Marzinke, Craig Hendrix, Souleymane Diabaté, Michel Alary

**Affiliations:** a Département de médecine sociale et préventive, Université Laval; b Centre de recherche du CHU de Québec-Université Laval, Québec, Canada; c Dispensaire IST, Cotonou, Bénin; d École Nationale de Formation des Techniciens Supérieurs en Santé Publique et en Surveillance Épidémiologique, Université de Parakou; e Faculté des sciences de la santé, Université d’Abomey-Calavi; f Programme Santé de Lutte contre le Sida (PSLS); g Centre national hospitalier universitaire HMK de Cotonou, Bénin; h Johns Hopkins University School of Medicine, Baltimore, MD, USA; i Université Alassane Ouattara, Bouaké, Côte d’Ivoire; j Institut national de santé publique du Québec, Québec, Canada.

**Keywords:** adherence, blood drug level, female sex workers, HIV prevention, PrEP, tenofovir

## Abstract

**Background:**

Measuring adherence to PrEP (pre-exposure prophylaxis) remains challenging. Biological adherence measurements are reported to be more accurate than self-reports and pill counts but can be expensive and not suitable on a daily basis in resource-limited countries. Using data from a demonstration project on PrEP among female sex workers in Benin, we aimed to measure adherence to PrEP and compare self-report and pill count adherence to tenofovir (TFV) disoproxil fumarate (TDF) concentration in plasma to determine if these 2 measures are reliable and correlate well with biological adherence measurements.

**Methods:**

Plasma TFV concentrations were analyzed in samples collected at day 14 follow-up visit and months 6, 12, 18, and 24 (or at last visit when follow-up was shorter). Self-reported adherence was captured at day 14 follow-up visit and then quarterly by asking participants to report the number of missed pills within the last week. For pill count, medications were refilled monthly and participants were asked to bring in their medication bottles at each follow-up visit. Using generalized estimating equations adherence measured by self-report and pill count was compared to plasma drug concentrations.

**Results:**

Of 255 participants, 47.1% completed follow-up. Weighted optimal adherence combining data from all visits was 26.8% for TFV concentration, 56.0% by self-report and 18.9% by pill count. Adherence measured by both TFV concentrations and self-report decreased over time (*P* = .009 and *P* = .019, respectively), while the decreasing trend in adherence by pill count was not significant (*P* = .087). The decrease in adherence was greater using TFV concentrations than the other 2 adherence measures.

**Conclusion:**

With high levels of misreporting of adherence using self-report and pill count, the objective biomedical assessment of adherence via laboratory testing is optimal and more accurately reflects PrEP uptake and persistence. Alternative inexpensive and accurate approaches to monitor PrEP adherence should be investigated.

## Abstract 2

Supplemental Digital Content is available in the text

## 1 Introduction

Daily oral pre-exposure prophylaxis (PrEP) is the use of antiretroviral drugs by high-risk seronegative individuals to prevent human immunodeficiency virus (HIV) acquisition. Several randomized controlled trials have shown that HIV PrEP efficacy among high-risk groups is dependent on good adherence.^[1–3]^ Indeed, studies of dosing patterns have shown a strong relationship between higher adherence and greater protective effect of daily tenofovir disoproxil fumarate (TDF)/ emtricitabine (FTC) (Truvada), the drug approved for PrEP.^[4,5]^ As countries progress toward implementation and scale-up, it is important to understand and support PrEP adherence beyond the context of clinical trials to maximize its public health impact.^[6]^

Measuring adherence to PrEP remains challenging and all methods are subject to bias or inaccuracy.^[7–9]^ Objective measures of adherence can be indirect like clinic-based pill counts, pharmacy refill frequency, electronic drug monitoring such as the medication event monitoring system or direct like pharmacologic measures such as plasma drug concentrations.^[10]^ Objective measures likely provide the most reliable data despite some drawbacks.^[6,10]^ For example, blood drug measurements are susceptible to manipulation since participants may take the medications prior to a planned blood draw. In addition, this method can be expensive and may not be feasible in resource limited settings. Pill count can also be susceptible to manipulation since individuals may remove pills from a medication bottle prior to the clinic visit to act like they were adherent. Subjective measures include physician or family reports and patients’ self-reports. Self-reports are inexpensive and easy to collect but can overestimate adherence due to social desirability and reporting biases.^[11,12]^ Hence, the choice of adherence measures is often based on feasibility and cost-effectiveness.^[13]^

Objective measures of adherence to PrEP (eg, electronic monitoring or drug concentration) are reported to be more accurate than self-reports and using multiple measures of adherence is more likely to increase accuracy in estimating adherence behaviour.^[9,14]^ However, for potential implementation of PrEP programs in resource limited countries, the most objective measures will be expensive and not suitable on a routine basis. It is thus necessary to determine wheter less expensive and more frequently used adherence measures are reliable and correlate well with biological adherence measurements.^[15]^ Finding the appropriate ways to measure adherence is essential to assure that the right guidance on how to support high adherence levels is developed for broader implementation and scaling-up. In this context, using data from a demonstration project on PrEP among female sex worker (FSW) in Benin, we conducted this study to measure adherence to PrEP among FSWs and compare self-report and pill count adherence to plasma drug concentrations

## 2 Methods

### 2.1 Study population

Data for this study were from the PrEP arm of a prospective demonstration study on early antiretroviral therapy (“test-and-treat”) and PrEP among professional and active FSWs in Benin (ClinicalTrials.gov NCT02237). PrEP was only available in the country for the purpose of the study. Study procedures are detailed elsewhere.^[16]^ Briefly, from October 2014 to December 2015, 256 professional FSWs (women whose revenue is mostly generated by sex work) were recruited in Cotonou and its inner suburbs and were followed until December 2016 at the Dispensaire IST. The PrEP participants were HIV-negative professional FSWs, ≥18 years old, had normal renal and liver functions, did not have active hepatitis B, were not pregnant or breastfeeding. FSWs were asked to take daily TDF/ emtricitabine (TDF/FTC, Truvada) and were first followed 14 days after the recruitment and later on a quarterly basis. Participants received their Truvada every month but could pick up a supply for up to 3 months if needed. Follow-up time varied from 12 to 24 months depending on the time of recruitment.^[16]^

### 2.2 Adherence assessment

Adherence to PrEP was assessed via measurement of tenofovir (TFV) in plasma, self-report and pill count. The terminal elimination half-life of TFV is approximately 17 (12.0–25.7) hours. Co-administration of Truvada with certain drugs to treat Hepatitis C virus increases TFV concentrations.^[17]^ TFV concentrations in plasma were analyzed in batch using a previously described liquid chromatographic-mass spectrometric assay. The lower limit of quantification (LLOQ) for TFV of the assay was 0.31 ng/mL. The assay was validated following US Food and Drug Administration bioanalytical recommendations.^[18]^ Drug measurements were conducted in samples collected at day 14 follow-up visit (D-14) and months 6, 12, 18 and 24 (or at the last visit for subjects with a shorter follow-up time). Based on directly observed therapy-based studies, the assay LLOQ can detect drug usage within the past week. Supplementary Table 1 shows the relationship between the number of pills taken in the last week and the TFV and FTC plasma concentration based on a 90% sensitivity as observed in a directly observed dosing study^[18]^ and that we used in the present study. Self-reported adherence was measured at D-14 and then every 3 months by asking participants during face-to-face interviews (FTFIs) to report the number of missed pills in the last week. For pill count, the medications were refilled every month and participants were asked to bring in their medication bottles at each follow-up visit. The number of unused pills between the medication dispensation date and the follow-up visit date was then recorded. Optimal daily adherence was defined as a TFV concentration ≥35.5 ng/mL, which was equivalent to taking all 7 pills in the last week by self-report or all 30 pills in the last month by pill count. Detectable adherence was defined as a TFV concentration ≥ 0.31 ng/mL, which was equivalent to ≥1 pill taken in the last week as measured by self-report or ≥4 pills taken in the last month as measured by pill count. Participants were unaware that drug measurements would be performed at some study visits to avoid influencing drug taking behavior. This condition, as well as the overall study, were approved by the ethics committee of the CHU de Québec-Université Laval and the National ethics committee in Benin.

### 2.3 Statistical analysis

Adherence proportions were calculated for each method for the follow-up visits where the 3 measurements were performed (D14, months 6, 12, 18, 24 or the last follow-up visit). These time periods were chosen to allow comparison between the 3 adherence measurements. We assessed the distribution of adherence based on TFV and FTC concentration as per the categories presented in supplementary Table 1. For self-report, adherence proportions were calculated by dividing the numbers of pills taken in the last week by the 7 days of the week. Pill count adherence proportions were calculated using the following equation: (number of pills dispensed minus number of remaining pills) divided by the elapsed days between the medication dispensation date and the follow-up visit date.

To define the analytical strategy for the comparison between the 3 types of adherence measurements and their time trends over the course of the study, we first examined the distribution of TFV and FTC across the pre-defined categories, that were very similar for both drugs (Supplementary Table 2). In addition, only 3.5% of all samples were discordant between TFV and FTC when using the LLOQ cutoff whereas the correlation coefficient between TFV and FTC concentration when detectable was very high at 0.9. Because of this, we only used the TFV concentration for our comparative and time trends analyses. Furthermore, as the vast majority of the samples (83.3%) were in the highest or lowest categories of adherence according to TFV plasma concentrations, we opted to carry out the comparative and time trend analyses using 2 cut-offs for the purpose of simplicity and statistical power. These cut-offs were defined as optimal and detectable adherence, respectively.

To calculate the proportions of optimal adherence using TFV drug concentrations, the number of participants with concentrations ≥35.5 ng/mL was divided by the total number of participants with TFV data measured at the follow-up visit. The same was done for participants who had detectable TFV concentrations (≥0.31 ng/mL). For each of the 3 adherence measures, results were categorized by 2 dichotomized variables, both (1) optimal (100%) or not optimal and (2) any detectable or no detectable adherence. Using binomial regression with generalized estimating equations assuming an unstructured working correlation structure to account for repeated measures across visits, adherence measured by self-report and pill count were compared to plasma drug concentration. Time trends in adherence from D-14 to month 24 were compared by simultaneously fitting a model for the 3 different adherence measurement methods. A similar model was fitted for time trends in adherence from D14 to month 12. To avoid potential selection bias due to high attrition because of late recruitments and withdrawals, the observed data were weighted by inverse probability of censoring (IPCW). IPCW inflates the impact of underrepresented subjects and diminishes that of overrepresented subjects so that observed estimates are representative of those that would have been observed if all subjects had stayed in the study. It thus allowed to reproduce a pseudo-population that would have been observed had losses to follow up occurred but been random with respect to measured determinants of loss to follow up. This pseudo-population was created by re-weighting the contribution of each participant who was not lost to follow up to a given set of predictors. For this, we first identified key predictors of participation at baseline. Then, for each of the follow-up visits (D-14, month 6 follow-up visit [M6], month 12 follow-up visit [M12], month 18 follow-up visit [M18] and month 24 follow-up visit [M24]), a logistic regression model was computed to generate the weights based on the covariates measured at the previous visit. For each of the logistic models, the assumptions of linearity, absence of multicollinearity, influential observations and quasi-complete separation were verified. The stabilized weight assigned to each participant corresponds to the inverse probability of that individual not being censored at that follow-up visit given the covariates measured at baseline and that the individual was not censored at the previous visit. Weights were then normalized by dividing them by their means as to not artificially inflate the sample size. Robust estimates of variance were used in the weighted models. The IPCW assumptions of exchangeability, positivity and no model misclassification were also verified.^[19,20]^ The calculated weights were then applied to run the generalized estimating equations models. All trends and comparisons between trends were analyzed using contrasts. Adherence estimates are reported with their 95% confidence interval (95%CI). Statistical analyses were performed using SAS 9.4 (SAS Institute Inc). Statistical significance was determined at *P* < .05.

## 3 Results

### 3.1 Participants characteristics

In the PrEP arm of the early antiretroviral therapy/PrEP study, 256 FSW were recruited.^[16]^ However, in our analysis, 1 participant was excluded for missing data on the variables used for the IPCW.

For the 255 (99.6%) FSWs included in the present study, mean age was 32.5 years (SD = 9.2). Close to half of the FSWs were Beninese (49.0%), 97.7% were not married and 65.9% did not complete secondary school. Mean duration (±SD) of follow-up was 11.8 ± 7.9 months.

### 3.2 Attrition

Out of the 255 participants at baseline, the number of included participants was 225 (88.2%) at D-14, 189 (74.1%) at M6, 151 (59.2%) at M12, 76 (29.8%) at M18 and 30 (11.8%) at M24. The number of attritions was 30 between baseline and D-14, 36 between D14 and M6, 38 between M6 and M12, 75 (53 were due to late recruitment and 22 to withdrawals) between M12 and M18 and 46 (37 were due to late recruitment and 9 to withdrawals) between M18 and M24. Thus, overall, 225 participants were not followed for 24 months either for late recruitment (N = 90) or withdrawals (N = 135) and 120 (47.1%) completed their follow-up. Reasons for withdrawal are reported elsewhere.^[16]^ The number of final visits completed for all recruited subjects was 151 (59.2%).

### 3.3 Adherence to PrEP assessed by the 3 different measures

#### 3.3.1 Optimal adherence

IPCW-weighted optimal adherence measured by TFV concentration varied from 50.2% (95%CI: 43.9–57.5) at D-14 to 14.9% (95%CI: 6.2–36.2) at M24 and global adherence (combining data from all visits) was 26.8% (95%CI: 20.8–34.6). For self-report, IPCW-weighted adherence varied from 77.6% (95%CI: 71.8–83.7) at D-14 to 46.0 (95%CI: 34.0–62.3) at M18 and then increased to 59.9% (95%CI: 42.1–85.4) at M24 and global adherence was 56.0% (95%CI: 49.3–63.6). The corresponding proportions according to pill count varied from 46.3% (95%CI: 39.3-54.7) at D-14 to 10.5% (95%CI: 4.3–25.8) at M18 and then increased to 30.8% (95% CI:12.1–78.6) at M24 and global adherence was 18.9% (95%CI: 13.5–26.4). Overall optimal adherence measured by self-report was about twice the adherence measured by TFV concentrations (prevalence ratio (PR): 2.1 (95%CI: 1.6–2.6).

#### 3.3.2 Detectable adherence

IPCW-weighted detectable adherence measured by TFV concentration varied from 71.3% (95%CI: 65.4-77.7) at D-14 to 28.5% (95% CI: 13.9-58.6) at M24, and global adherence was 40.9% (95%CI: 32.4-51.6). For self-report, it varied from 95.3% (95% CI: 92.7-98.4) at D14 to 60.2% (95% CI: 47.9-75.7) at M18, then increased to 70.6% (95% CI: 53.9-92.5) at M24, whereas global adherence was 73.1% (95%CI: 66.7-80.0). By pill count, it varied from 99.4% (95% CI: 98.5-100) at D-14 to 61.6% (95% CI: 34.9-100) at M24, and global adherence was 77.3% (95%CI: 68.4-87.3). Prevalence ratios comparing detectable adherence measured by self-report and by pill count to adherence measured by TFV concentrations were respectively 1.8 (95%CI: 1.4-2.2) and 1.9 (95%CI: 1.4-2.5). Adherence measured by pill count was similar to adherence measured by self-report (PR = 1.1 (95%CI: 0.9-1.2)).

### 3.4 Comparison of trends in adherence

#### 3.4.1 Trends over 24 months

**Figure 1:**
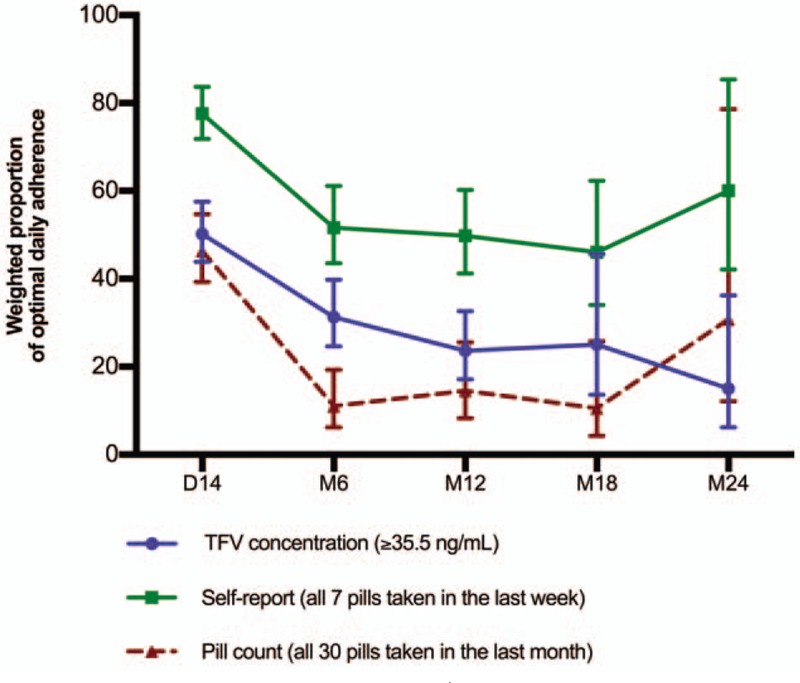
Weighted proportion of optimal^∗^ daily adherence (100%) to PrEP measured by TFV blood concentration, self-report and pill count in the PrEP demonstration study conducted among female sex workers in Cotonou, Benin. TFV: Tenofovir; PrEP: pre-exposure prophylaxis; D-14 and M6, M12, M18, M24: d 14 and mo 6, 12, 18, and 24 follow-up visits; GEE: Generalized estimating equations. Vertical bars denote 95% confidence intervals (CI); ^∗^Optimal adherence means that the participant had a TFV concentration ≥ 35.5 ng/mL which was equivalent to taking all 7 pills in the last week as measured by self-report or all 30 pills in the last month as measured by pill count. The number of individuals contributing data at D-14, M6, M12, M18, and M24 is respectively 225, 189, 151, 76, and 30. The observed data were weighted by probability of censoring (IPCW) because of high attrition due to late recruitment and withdrawals. Follow-up time varied from 12 to 24 mo depending on the time of recruitment. Self-reported adherence was measured at D-14 and then quarterly. Pill count was performed every month and TFV concentration was measured on samples collected at D-14 and M6, M12, M18, M24. Comparisons were done for the follow-up visits where the 3 measures were performed. p-trend over 24 months for TFV concentration = .007; *P*-trend over 24 mo for self-report = .162; *P*-value for the comparison of the trend assessed by TFV concentration to the trend assessed by self-report over 24 mo = .037; Test of trends from D-14 to M24 assessed by contrast using GEE. A total of 225 women contributed to 1516 observations. *P*-trend over 12 months for TFV concentration = < .001; *P*-trend over 12 mo for self-report = < .001; *P*-value for the comparison of the trend assessed by TFV concentration to the trend assessed by self-report over 12 mo = .063; Test of trends from D-14 to M12 assessed by contrast using GEE. A total of 225 women contributed to 1274 observations. For optimal adherence, pill count is not comparable to the other adherence measures because the measures are not always aligned due to differences in the reporting periods. For this matter, no statistical tests are reported for pill count and no comparisons between pills count measures and TFV concentration and self-report were performed.

**Figure 2:**
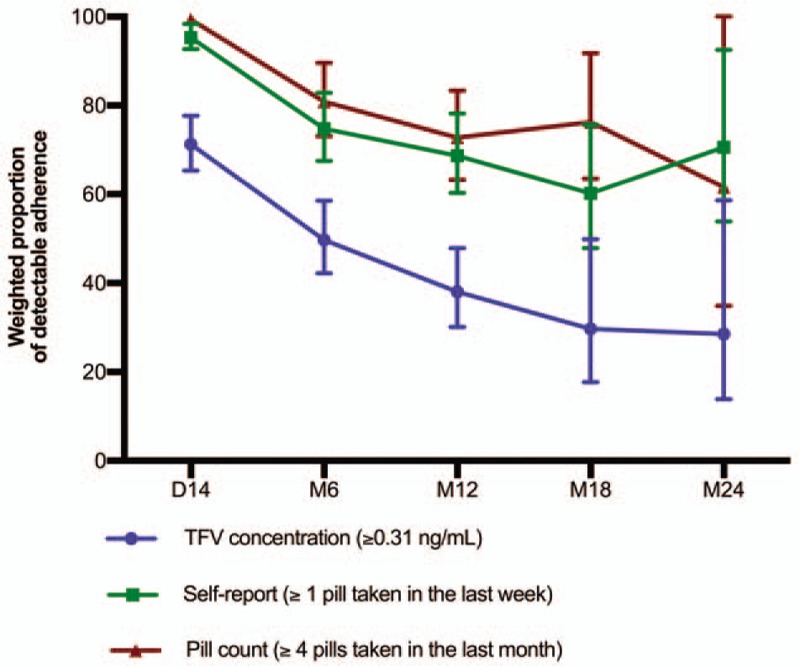
Weighted proportion of detectable^∗^ adherence to PrEP measured by Tenofovir (TFV) blood concentration, self-report and pill count in the PrEP demonstration study conducted among female sex workers in Cotonou, Benin. TFV: Tenofovir; PrEP: pre-exposure prophylaxis; D-14 and M6, M12, M18, M24: d 14 and mo 6, 12, 18 and 24 follow-up visits; GEE: generalized estimating equations. Vertical bars denote 95% confidence intervals (CI) ^∗^Detectable adherence means that the participant had a TFV concentration ≥ 0.31 ng/mL which was equivalent to taking all ≥ 1 pill in the last week as measured by self-report or ≥ 4 in the last month as measured by pill count. Follow-up time varied from 12 to 24 mo depending on the time of recruitment. The number of individuals contributing data at D-14, M6, M12, M18, and M24 is respectively 225, 189, 151, 76, and 30. The observed data were weighted by probability of censoring (IPCW) because of high attrition due to late recruitment and withdrawals. Self-reported adherence was measured at D-14 and then quarterly. Pill count were performed every month and TFV concentration was measured on samples collected at D-14 and M6, M12, M18, M24. Comparisons were done for the follow-up visits where the 3 measures were performed. *P*-trend over 24 mo for TFV concentration = .009; *P*-trend over 24 mo for self-report = .019; *P*-trend over 24 mo for pill count = .087; *P*-value for the comparison of the trend assessed by TFV concentration to the trend assessed by self-report over 24 mo = .058; *P*-value for the comparison of the trend assessed by TFV concentration to the trend assessed by pill count over 24 mo = .267; *P*-value for the comparison of the trend assessed by self-report to the trend assessed pill count over 24 mo = .767; Global comparison of trends over 24 mo of follow-up: *P* = .115 (Test with 2 degrees of freedom to simultaneously compare the trends in adherence between the 3 measures); Test of trends from D-14 to M24 assessed by contrast using GEE. A total of 225 women contributed to 1516 observations. *P*-trend over 12 mo for TFV concentration = < 0.001; p-trend over 12 mo for self-report = < .001; *P*-trend over 12 mo for pill count = < .001; *P*-value for the comparison of the trend assessed by TFV concentration to the trend assessed by self-report over 12 mo = .005; *P*-value for the comparison of the trend assessed by TFV concentration to the trend assessed by pill count over 12 mo = .017; *P*-value for the comparison of the trend assessed by self-report to the trend assessed by pill count over 12 mo = .861; Global comparison of trends over 12 mo of follow-up: *P* = .017 (Test with 2 degrees of freedom to simultaneously compare the trends in adherence between the 3 measures); Test of trends from D-14 to M12 assessed by contrast using GEE. A total of 225 women contributed to 1274 observations. Comparisons between pill count measures and self-report and TFV concentration are feasible since the cut-off classified as detectable measures any dosing above the lower limit of quantification.

Trends in adherence are presented in Figure 1 for optimal adherence and Figure 2 for detectable adherence. Optimal adherence by TFV concentrations decreased over the course of the study (*P*-trend = .007) and adherence by self-report also decreased although the test of trend was not significant (*P*-trend = .162). Trends in adherence measured by TFV concentrations were different from trends in adherence measured by self-report (*P* = .037). For pill count, statistical tests were not performed because the measures between the 3 methods are not always aligned due to differences in the reporting periods (Fig. 1).

Detectable adherence measured by TFV concentrations and self-report decreased over the 24 months of follow-up (*P* = .009 and *P* = .019, respectively) and adherence by pill count decreased but the trend was not significant (*P* = .087). There was no significant difference in the trends between TFV and self-report or pill count (*P* = .058 and p = .267, respectively). The decrease in adherence was however greater using TFV concentrations than the other 2 adherence measures (Fig. 2).

#### 3.4.2 Trends over 12 months

Weighted optimal adherence measured by TFV concentrations and self-report decreased over the first 12 months of follow-up (*P* < .001) and there was no significant difference in trends between the 2 methods (*P* = .063) (Fig. 1). Weighted detectable adherence decreased over the first 12 months of follow-up decreased for all 3 adherence measures (*P* < .001, Fig. 2). The trend in adherence as measured by TFV concentrations was different from the trend in adherence as measured by self-report (*P* = .005) and by pill count (*P* = .017) over the first 12 months of study.

### 3.5 Comparison of adherence at baseline (D14) and final visits

Tables 1 and 2 present weighted proportions of optimal daily adherence and detectable adherence to PrEP at baseline (D-14) and at all final visits (withdrawals or visits due to the end of the study), respectively. For the 3 measures, optimal adherence and detectable adherence were less at final visits compared to D-14 (*P* < .001). Sensitivity analyses were performed for comparison of adherence at D-14 and final visits due to the end of the study and the results were similar (data not shown). Results were also similar when the analysis was restricted to participants who had both D-14 and final visit data (data not shown).

**Table 1:**
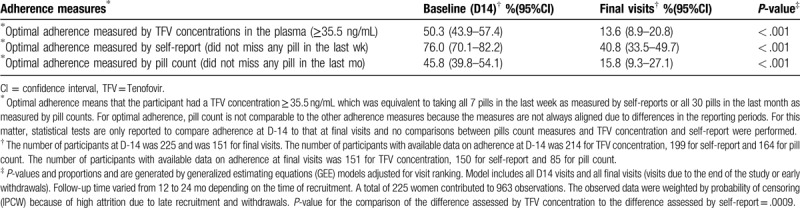
Comparison of weighted proportions of optimal daily adherence to PrEP at baseline (d 14) and at all final visits measured by 3 different methods in the E-ART-PrEP demonstration project in Cotonou, Benin.

**Table 2:**
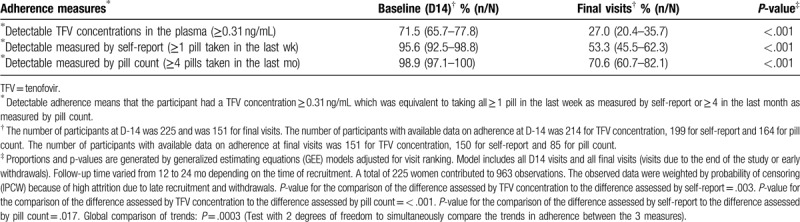
Comparison of weighted proportions of detectable adherence to PrEP at baseline (day 14) and at all final visits measured by 3 different methods in the E-ART-PrEP demonstration project in Cotonou, Benin.

## 4 Discussion

Using data from the demonstration project on daily HIV PrEP conducted among FSWs in Benin, we described and compared patterns of adherence using TFV concentrations in plasma, self-report and pill counts. We found that optimal daily adherence (no pills missed in the reporting period) was low to moderate using all 3 measures. By contrast, detectable adherence (TFV concentration ≥ 0.31 or having taken 1 or more pills in the last week using self-report or 4 or more pills using pill counts) was higher. For both the optimal and detectable adherence definitions, adherence decreased over the course of the study as shown by all 3 measures. When we limited the analysis to the first 12 months of study, decrease in adherence was more profound. Overall, even though adherence waned during study duration using all 3 measures, measures using self-report and pill count were generally higher than measures using TFV plasma measurements.

These findings on decrease in adherence throughout the study are consistent with results from PrEP demonstration projects among men who have sex with men (MSM)^[21]^ where adherence decreased with long-term participation. Indeed, in this MSM study, the majority of participants had TFV levels consistent with ≥4 pills/week over the first 12 weeks of the study, a noticeable drop-off occurred at week 24, and by week 48, only 34% of participants had this level of drug detected.^[21]^ Among FSWs, the TAPS study, conducted in South Africa, only provide data on self-reported adherence to PrEP. It showed higher adherence rates than our study, with a slight decrease over time: 85% after 3 months of follow-up, 70% after 9 months of follow-up and 81% after 21 months of follow-up.^[22]^

Despite the decrease in adherence in demonstration studies, PrEP adherence has generally been higher in recent trials, open-label extensions, and demonstration projects compared to the initial clinical trials.^[23]^ The difference we found between adherence assessed through self-report or pill-count and adherence assessed through drug concentrations in plasma (or other biological samples) was also reported in other studies.^[24]^ For example, in the VOICE study, a PrEP trial conducted among high-risk women in South Africa, Uganda and Zimbabwe, adherence to the pills or microbicide was 93%, by pill count and self-report, but only 28% to 29% of participants assigned to the study drug and 22% of participants assigned to the TFV 1% vaginal gel had detectable drug concentrations in their blood.^[1,24]^ The iPrEx trial, another PrEP study conducted among MSM and transgender women in six countries, reported that adherence measured by pill count and self-report was > 90%,but only 51% by blood drug levels.^[25]^

Self-report is generally reported to correlate poorly with adherence as determined by drug concentration in plasma.^[1,3]^ In our study, we found similar results, as self-reported adherence overestimated adherence measured by TFV concentrations by a factor of 2. However, we observed the same decreasing trend in adherence persistence using self-report and TFV plasma concentrations. This overestimation can be attributed to social desirability and recall biases, especially when we assessed optimal adherence. Indeed, in our study, participants were regularly counselled on the importance of adherence for PrEP effectiveness. In this case, participants may have feared being judged if they reported suboptimal levels of adherence. For this matter, we also evaluated a less stringent cut-off of adherence (detectable vs undetectable) to compare adherence using self-reports and using TFV concentrations. We found that even though adherence using self-report overestimated adherence compared to TFV plasma concentrations, there was no difference in the trends over 24 months of follow-up. This finding suggests that self-reports of imperfect adherence can be trusted in our study despite the overestimation. We used FTFIs in this study because on their ease of use and low-cost. However, the literature suggests that other modes of self-report offer more privacy and are therefore less affected by social desirability biases. These modes include self-administered questionnaires and computerized interviews such as ACASI (Audio computer-assisted self-interview).^[26,27]^ Given the research context of a resource limited country like Benin and the level of literacy of our study population, we chose FTFIs for data collection. Moreover, in the VOICE study, ACASI did not improve the accuracy of self-reported product use compared with FTFI, despite previous research indicating that ACASI is preferred by participants and may increase reports of sensitive behavior.^[11]^

We also found that although detectable adherence using pill count overestimated adherence measured using TFV concentration, there was no difference in the trends. Both measures showed a decrease in adherence during the study period. We believe the difference between the 2 measures was primarily due to social desirability bias. Indeed, participants may have removed pills from the bottle to give appearance of adhering.^[4,28]^ In our study, some participants reported transferring all their pills from the medication bottles to plastic bags to avoid stigmatization. They were afraid to be seen by their entourage (peers, family members, regular partners, and so on) with a medication known to treat HIV. Another explanation for the misclassification of adherence using pill count could be pill sharing. However, participants were told that the medication was only for their personal use. Additionally, many participants did not bring back their medication bottles for pill count during study visits, which also led to a higher number of missing values for pill count compared to other adherence measures. We thus believe that adherence measures by pill count largely overestimated the actual adherence levels of the FSWs.^[29]^ A qualitative study to understand the reasons for low adherence was conducted and results will be presented in a subsequent publication.

When we measured detectable adherence, we observed significant differences in the trends comparing adherence measured using self-report and adherence measured using TFV concentrations and also between adherence measured using pill count and adherence measured using TFV concentrations after 12 months of follow-up. However, this difference in trends was not seen after 24 months of follow-up. The reasons why were observed this difference in the comparison on trends after 12 months and 24 months of follow-up can be explained by the fact that the participants who overestimated their adherence level were in reality less adherent and tended to leave the study before the end. Recent research has shown that short-term objective adherence is strongly associated with retention in the study in a secondary data analysis of a large, prospective multi-site demonstration project among MSM and transgender women.^[30]^

This study has several limitations. First, we observed a high level of attrition because of late recruitments and withdrawals throughout the study. To correct for a potential selection bias due to the importance of missing data, data were weighted by inverse probability of censoring.^[31]^ Although we tried to correct for the potential selection bias, we cannot exclude the possibility that the correction was imperfect. Second, the high attrition we observed resulted in a decrease in statistical power over the course of the study, which has probably affected some tests for trends. Third, the approach we have used to compare self-report, pill count and TFV concentration may be limited by the differences in time period for assessing adherence by the 3 different measures. Indeed, pill count could not be compared to the other measures for optimal adherence due to the difference in the reporting period. However, we used a second threshold to allow us to make appropriate comparisons. Fourth, TFV concentration in plasma reflects a short window prior drug dosing (up to 7 days prior to sample collection) and can also be susceptible to intra-individual variability and “white coat” adherence (a participant who only took medications shortly before sample collection could achieve plasma concentrations similar to those of participants who adhered consistently).^[24]^ Research has shown that longer term (6–8 weeks) adherence to PrEP can be measured using TFV diphosphate in dried-blood specimen.^[32]^ However, our objective was to compare adherence measured by self-report to adherence measured by TFV concentrations and thus measuring long term adherence was not relevant for our study. Fifth, even though we used various methods to improve the reliability of participants’ responses (e.g. approaching adherence questions with participants in a nonjudgmental manner), adherence measures by self-report and pill count were still subject to social desirability bias. Lastly, our results might not be generalizable to other populations.

In conclusion, with such high levels of misreporting of PrEP adherence using self-report and pill count found in our study, testing for the presence of TFV in blood remains the measure of choice despite its cost and invasiveness. However, the recent developments towards a urine point-of-care test for TFV measurement could eventually overcome these problems.^[33]^ Urine TFV concentrations can inform interpretation of novel point-of-care urine-based TFV assays to assess recent TDF adherence.^[34]^ It has also been recently reported that direct quantification of TFV in human blood using miniature mass spectrometry was a promising simple, fast and cost-effective method to monitor PrEP.^[35]^ Such alternative cheap and accurate biological approaches to monitor adherence should be further investigated for their practical use in clinical settings.

## Acknowledgments

We thank all the participants and team members in Benin and Canada. The views expressed herein are those of the authors and do not necessarily reflect the official policy or position of the Bill and Melinda Gates Foundation.

## Author contributions

MA and LB conceived the study. NG, DMZ, FG, KG, MM, CH, SD, FG, MB, RKK, DA and AM helped with the study conception. MM and CH performed plasma TFV analysis. LB managed the data and AM performed data analysis. AM drafted the manuscript. All authors revised the manuscript for important intellectual content and read and approved the final manuscript.

## Supplementary Material

Supplemental Digital Content
