# Supplementary material for: Comparison of adherence measurement tools used in a pre-exposure prophylaxis demonstration study among female sex workers in Benin
Source: Medicine (Baltimore). 2020 May 22;99(21):e20063. doi: 10.1097/MD.0000000000020063 (PMC7249870; doi:10.1097/MD.0000000000020063)
Supplement: Supplemental Digital Content [file medi-99-e20063-s001.docx]

**Supplementary information**

**Comparison of adherence measurement tools used in a pre-exposure prophylaxis demonstration study among female sex workers in Benin**

Aminata MBOUP, Luc BÉHANZIN, Fernand GUÉDOU, Katia Giguère, Nassirou GERALDO, Djimon Marcel ZANNOU, René K. KÊKÊ, Moussa BACHABI, Flore GANGBO, Dissou AFFOLABI, Mark A. MARZINKE, Craig HENDRIX, Souleymane DIABATÉ, Michel ALARY

Table 1. Relationship between the number of pills taken in the last week and the tenofovir (TFV) and emtricitabine (FTC) plasma concentrations

|  | **7 pills taken in the last week** | **4 pills taken in the last week** | **2 pills taken in the last week** | **1 pill taken in the last week** | **Below quantification limit** |  |
| --- | --- | --- | --- | --- | --- | --- |
| **FTC concentration in ng/mL** | | ≥ 35.4 | 4.2-35.4 | 2.5-4.1 | 0.31-2.4 | <0.31 |
| **TFV concentration in ng/mL** | | ≥ 49.1 | 4.6-49. | 3.8-4.5 | 0.31-3.7 | <0.31 |

Table 2. Unweighted adherence based on tenofovir (TFV) or emtricitabine (FTC) plasma concentrations at the different study visits

| **Adherence level based on TFV or FTC concentration** | | **D14***  **(N=214)**  **N (%)** | **M6***  **(N=150)**  **N (%)** | **M12**  **(N=124)**  **N (%)** | **M18**  **(N=51)**  **N (%)** | **M24**  **(N=30)**  **N (%)** | **Overall***  **(N=569)** |
| --- | --- | --- | --- | --- | --- | --- | --- |
| **7 pills per week** | **TFV: ≥ 35.4 ng/mL** | 109 (50.9) | 48 (32.0) | 31 (25.0) | 11 (21.6) | 6 (20.0) | 205 (36.0) |
|  | **FTC: ≥ 49.1 ng/mL** | 110 (51.6) | 49 (32.9) | 33 (26.6) | 11 (21.6) | 6 (20.0) | 209 (36.8) |
| **4 pills per week** | **TFV :4.2-35.4 ng/mL** | 26 (12.1) | 15 (10.0) | 10 (8.0) | 1 (1.9) | 1 (3.3) | 53 (9.3) |
|  | **FTC: 4.6-49.0 ng/mL** | 27 (12.7) | 17 (11.4) | 11 (8.9) | 1 (1.9) | 2 (6.7) | 58 (10.2) |
| **2 pills per week** | **TFV: 2.5-4.1 ng/mL** | 3 (1.4) | 1 (0.7) | 2 (1.6) | 0 (0.0) | 1 (3.3) | 7 (1.2) |
|  | **FTC: 3.8-4.5 ng/mL** | 1 (0.8) | 0 (0.0) | 0 (0.0) | 0 (0.0) | 0 (0.0) | 1(0.2) |
| **1 pill per week** | **TFV: 0.31-2.4 ng/mL** | 16 (7.5) | 11 (7.33) | 6 (4.8) | 2 (3.9) | 0 (0.0) | 35 (6.1) |
|  | **FTC: 0.31-3.7 ng/mL** | 15 (7.0) | 6 (4.0) | 5 (4.0) | 3 (5.9) | 1 (3.3) | 30 (5.3) |
| **Below quantification level** | **TFV: <0.31 ng/mL** | 60 (28.0) | 75 (50.0) | 75 (60.5) | 37 (72.6) | 22 (73.3) | 269 (47.3) |
|  | **FTC: <0.31 ng/mL** | 60 (28.2) | 77 (51.7) | 75 (60.5) | 36 (70.6) | 21 (70.0) | 269 (47.4) |

TFV=tenofovir

FTC=emtricitabine

* One missing data for FTC concentration at Day 14 (N=213) and at month 6 visit (N=149). Overall, for FTC, there was 2 missing data (N=567).
